# Formation of albumin manganese carbonate hybrid microparticles by confined impinging jet mixing with a tubular maturation section

**DOI:** 10.3762/bjnano.17.94

**Published:** 2026-10-07

**Authors:** Cinzia-Paulina Draeger, Yu Xiong, Saranya Chaiwaree, Radostina Georgieva, Anja Drews, Hans Bäumler

**Affiliations:** 1 Institute of Transfusion Medicine, Charité-Universitätsmedizin Berlin, 10117 Berlin, Germany; 2 HTW Berlin – University of Applied Sciences, School of Life Science Engineering, Wilhelminenhofstraße 75A, 12459 Berlin, Germany; 3 Department of Medical Physics, Biophysics and Radiology, Medical Faculty, Trakia University, Stara Zagora 6000, Bulgaria; 4 Department of Pharmaceutical Technology and Biotechnology, Faculty of Pharmacy, Payap University, Chiang Mai 50210, Thailand

**Keywords:** confined impinging jet mixer, continuous-flow synthesis, encapsulation efficiency, human serum albumin, hybrid microparticles, manganese carbonate

## Abstract

Hybrid protein inorganic microparticles are promising carriers for controlled drug delivery because their size, composition, and surface chemistry can be tailored to therapeutic requirements. In this study, human serum albumin–manganese(II) carbonate hybrid microparticles (MPs) were synthesized for the first time by continuous coprecipitation in a confined impinging jet mixer (CIJM) equipped with a tubular maturation section. Equimolar precursor solutions containing 10 mg·mL^−1^ HSA were mixed at total flow rates between 2 and 20 mL·min^−1^, and mean maturation residence times of 0, 6, and 12 s were achieved using capillaries of defined length. Particle characteristics were quantified by dynamic light scattering, laser Doppler electrophoresis, confocal laser scanning microscopy, and UV–vis spectrophotometry. Increasing flow rate reduced both the Kolmogorov length scale and the MPs’ hydrodynamic diameter, yielding MPs of 240–800 nm with polydispersity indices below 0.25. In contrast, residence time did not significantly affect size but increased the encapsulation efficiency from 43% to 65% and shifted the zeta potential to more negative values, indicating enhanced protein incorporation. These results show that CIJM hydrodynamics determine MP size, while the maturation section provides an independent lever to tune loading and surface properties, offering a robust route for engineering hybrid MPs for biomedical applications.

## Introduction

Since the 1970s, micro- and nanomaterials have enabled transformative advances in nanomedicine by providing carriers for targeted drug delivery, controlled release, and theranostic applications [1–2]. Microparticles (MPs) can prolong systemic circulation, protect labile cargos, and decouple dosing from immediate pharmacokinetics, features crucial for treating chronic and complex diseases such as cancer, diabetes, and neurodegenerative disorders [2–5]. Consequently, organic–inorganic hybrid MPs continue to be central to biomedical and pharmaceutical research [1–2,6–11].

Particle size and size distribution critically influence biodistribution, cellular uptake, drug release, and overall bioavailability [3–5]. While smaller particles generally enhance cellular internalization and tissue penetration, larger MPs can provide improved colloidal stability and extended release profiles, especially in depot formulations [3–5]. Hybrid systems combining organic matrices (e.g., proteins and polymers) with inorganic phases further expand the design space by enabling co-tuning of size, morphology, porosity, and surface chemistry [2,8,12–14].

A prototypical hybrid system is manganese(II) carbonate (MnCO_3_) precipitated in the presence of human serum albumin (HSA) [8,12]. MnCO_3_ formation from supersaturated MnCl_2_/Na_2_CO_3_ solutions is driven by its low solubility product. Once supersaturation exceeds the equilibrium solubility, nucleation and subsequent crystal growth lead to microparticle formation [15]. Proteins strongly modulate mineral precipitation: Under homogeneous conditions, nuclei form spontaneously once local supersaturation is reached; in heterogeneous systems, proteins act as nucleation templates, lowering the activation barrier and directing crystal morphology [13–14,16]. For HSA–MnCO_3_ hybrid MPs, homogeneous and heterogeneous nucleation pathways act synergistically, enabling controlled crystal architectures [12–14]. During maturation, decreasing supersaturation and ion diffusion towards crystal surfaces govern growth rate, defect density, and hierarchical structure [13–14,17–20].

Modern continuous precipitation technologies such as flash nanoprecipitation and microreactor platforms offer precise control over supersaturation history, mixing time, and residence time, thereby enabling narrow size distributions and adjustable internal architectures [21–24]. However, they suffer from low throughput, clogging issues, and a lack of controlled maturation times required for hybrid particle assembly. Confined impinging jet mixing (CIJM) is particularly attractive because the collision of high-velocity jets generates intense micromixing and rapid supersaturation in a small volume [21,25–26].

CIJM enables high-throughput, scalable production of MPs and nanoparticles with tight control over hydrodynamics and mixing time [21,26–27]. The flow field is governed primarily by the Reynolds number, Re (Equation 1 in the Methods section), of the colliding jets. Experimental and numerical studies indicate that Re ≈ 150–200 is sufficient to generate strong mixing, whereas Re ≥ 600 yields fully developed turbulent micromixing, which is favorable for uniform particle formation [25,28–30]. At the smallest scales, turbulent mixing is characterized by the Kolmogorov length scale, η [27–28]. Downstream of the mixing chamber, a tubular maturation section can provide a well-defined residence time for particle growth, ripening, and restructuring under laminar flow, but also raise the risk of aggregation, depending on the flow regime and colloidal stability [31–35]. Previous studies on particle formation via CIJM have largely focused on organic nanoparticles such as liposomes and lipid nanoparticles, whereas the production of protein–inorganic hybrid MPs using CIJM with a maturation section in continuous synthesis remains insufficiently explored [21,36–39].

In this study, HSA–MnCO_3_ hybrid MPs were synthesized for the first time by continuous coprecipitation in CIJM equipped with a tubular maturation section. The influence of the flow rate via its effect on Re, η, and mixing intensity on the size and PDI of HSA–MnCO_3_ hybrid MPs and the effects of residence time in a tubular maturation section at fixed flow rate on particle size, PDI, zeta potential, and HSA encapsulation efficiency were investigated. By combining hydrodynamic analysis with detailed physicochemical characterization, we aim to provide a process engineering framework for scalable, reproducible synthesis of HSA–MnCO_3_ hybrid MPs suitable for drug delivery applications.

## Results and Discussion

### Fluid properties and hydrodynamic regime

Table 1 presents the rheological properties of the precursor solutions (0.25 M Na_2_CO_3_; 0.25 M MnCl_2_ containing 10 mg·mL^−1^ HSA) and the resulting HSA–MnCO_3_ suspension. The kinematic viscosities at 22.8 °C of all fluids are only slightly higher than that of water at the same temperature (≈8.95 × 10^−7^ m^2^·s^−1^). Despite the presence of colloidal particles, no shear-thinning or viscoelastic effects were observed. The low solid fraction of about 3 wt % and modest interparticle interactions are consistent with the Newtonian approximation for hard-sphere suspensions at low volume fractions [40].

**Table 1:** Measured and estimated material properties (*n* = 3, *T* = 22.8 °C).

Fluid	Density ρ_f_ [kg·m^−3^]	Kinematic viscosity ν_f_ [m^2^·s^−1^]

0.25 M Na_2_CO_3_	1006.73 ± 0.01	9.15 × 10^−7^ ± 2.98 × 10^−9^
0.25 M MnCl_2_ with 10 mg·mL^−1^ HAS	1008.83 ± 0.01	9.34 × 10^−7^ ± 5.95 × 10^−9^
HSA-MnCO_3_ suspension	1007.20 ± 0.01	9.30 × 10^−7^ ± 3.97 × 10^−9^
arithmetic mean^a^	1007.59 ± 1.10	9.27 × 10^−7^ ± 1.00 × 10^−8^

^a^To describe the mixed state in the IJM chamber (assumption: ideally mixed with ρ_f_ = constant). These properties were used as input for all subsequent calculations of Reynolds number, Re, energy dissipation rate, ε, and Kolmogorov length scale, η*.*

### Influence of flow rate on microparticle formation

**Figure 1:**
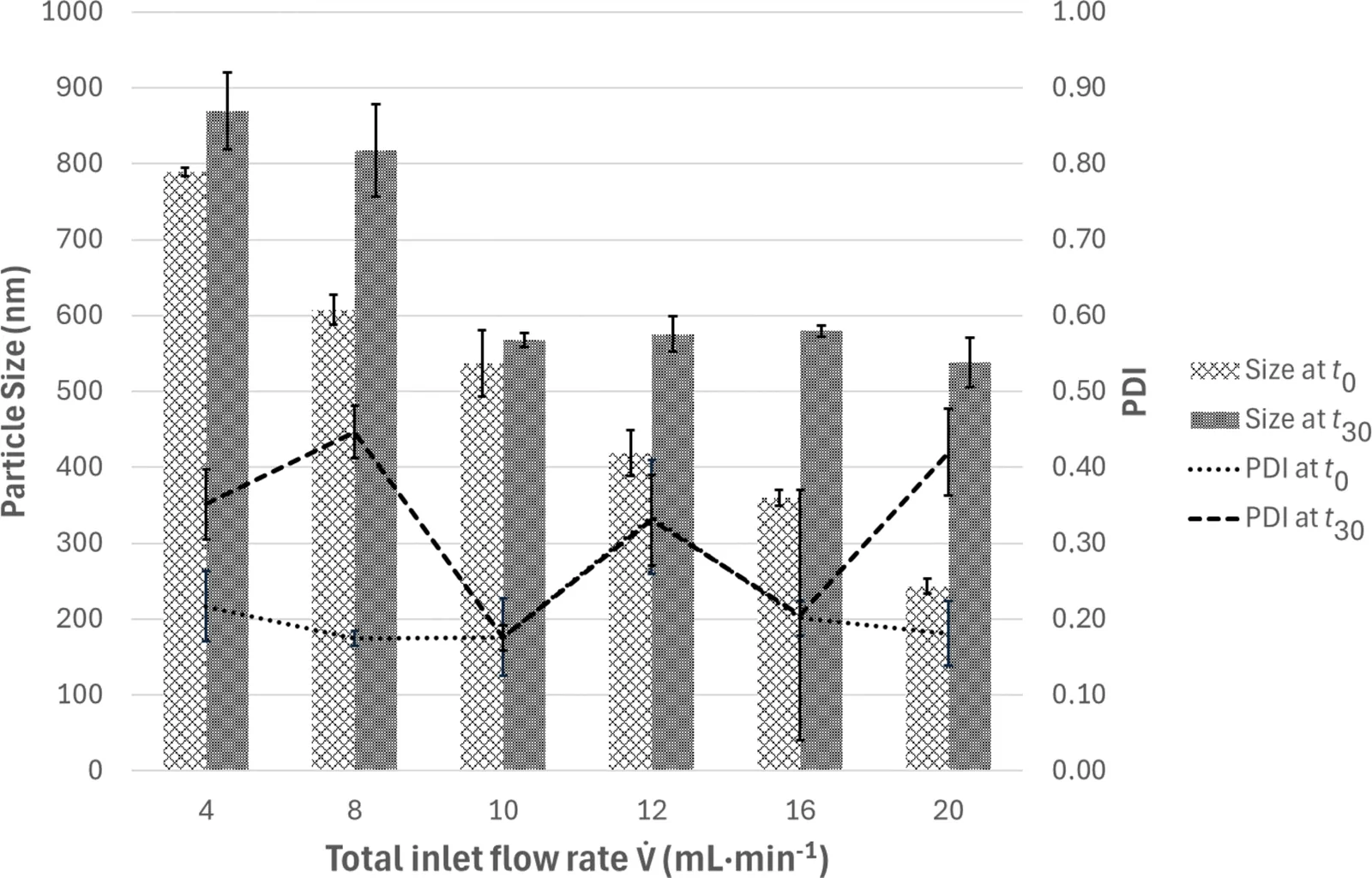
Particle size and PDI as functions of the total inlet flow rates, measured immediately after collection (*t*_0_) and after 30 min of incubation following collection (*t*_30_) (*n* = 3).

The particle sizes of the samples at different flow rates were measured immediately after collection (*t*_0_) and after 30 min of incubation following collection (*t*_30_). As shown in Figure 1, the size of the hybrid particles at *t*_0_ decreased from approximately 800 nm at a total inlet flow rate of 4 mL·min^−1^ to approximately 250 nm at 20 mL·min^−1^, with the polydispersity indexes (PDI) in the range of 0.2–0.3. However, particle sizes stabilized after 30 min of incubation following collection.

**Figure 2:**
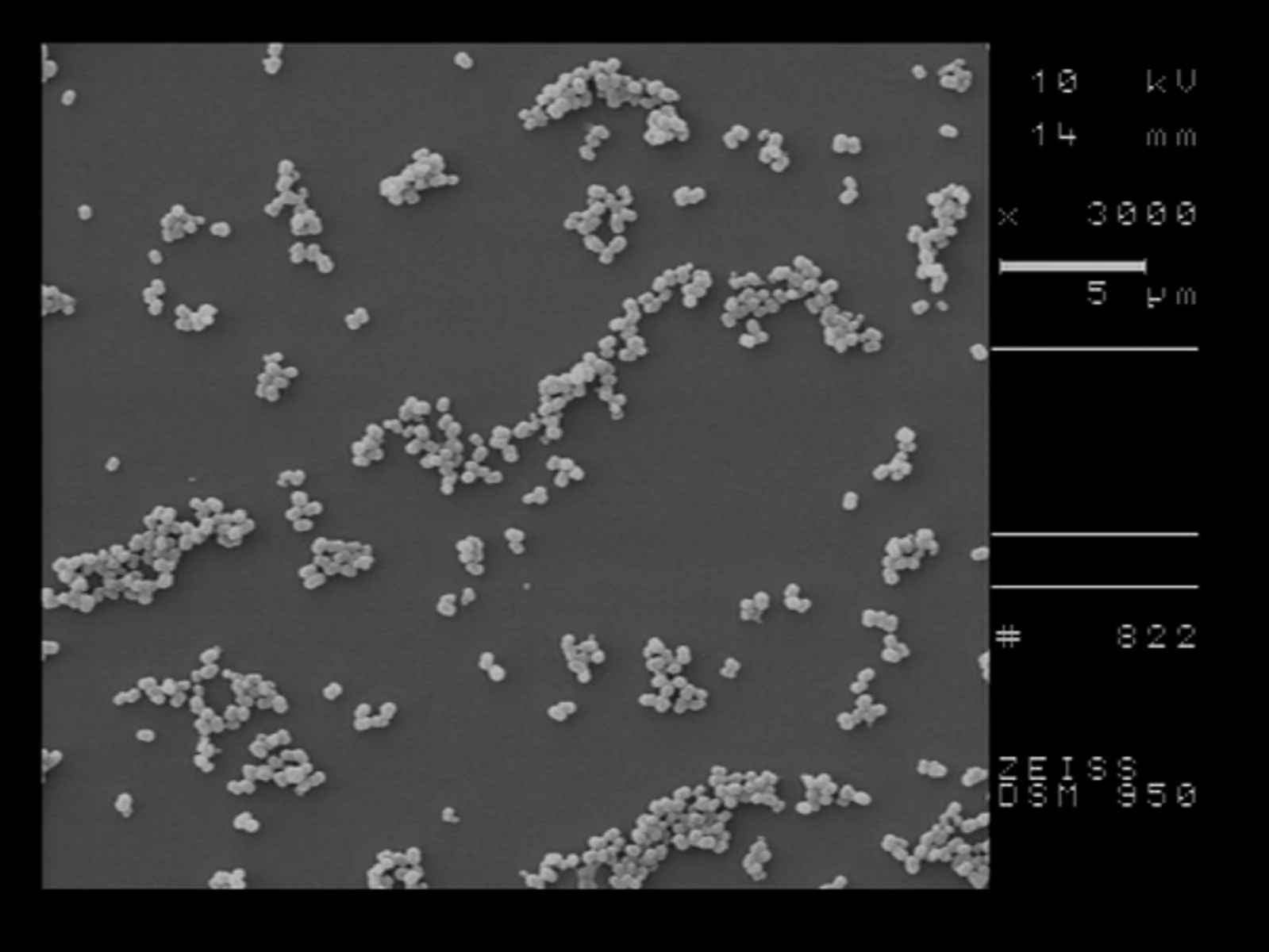
Scanning electron microscopy (SEM) image of HSA–MnCO_3_ hybrid particles at total inlet flow rates of 10 mL·min^−1^.

While particles at total inlet flow rates of 4 mL·min^−1^ and 8 mL·min^−1^ grew to over 800 nm at *t*_30_, particle sizes in the experiments conducted at total inlet flow rates of 10 mL·min^−1^ and higher remained between 540 and 600 nm without any significant dependence on flow rate. The PDIs at *t*_30_ were in the range of 0.2–0.4. Under experimental conditions, the nanoparticles continue to grow into microparticles, which corresponds to the normal crystal growth process [14–15,41]. As viewed by scanning electron microscopy, the particles are spherical or peanut-shaped (Figure 2).

**Figure 3:**
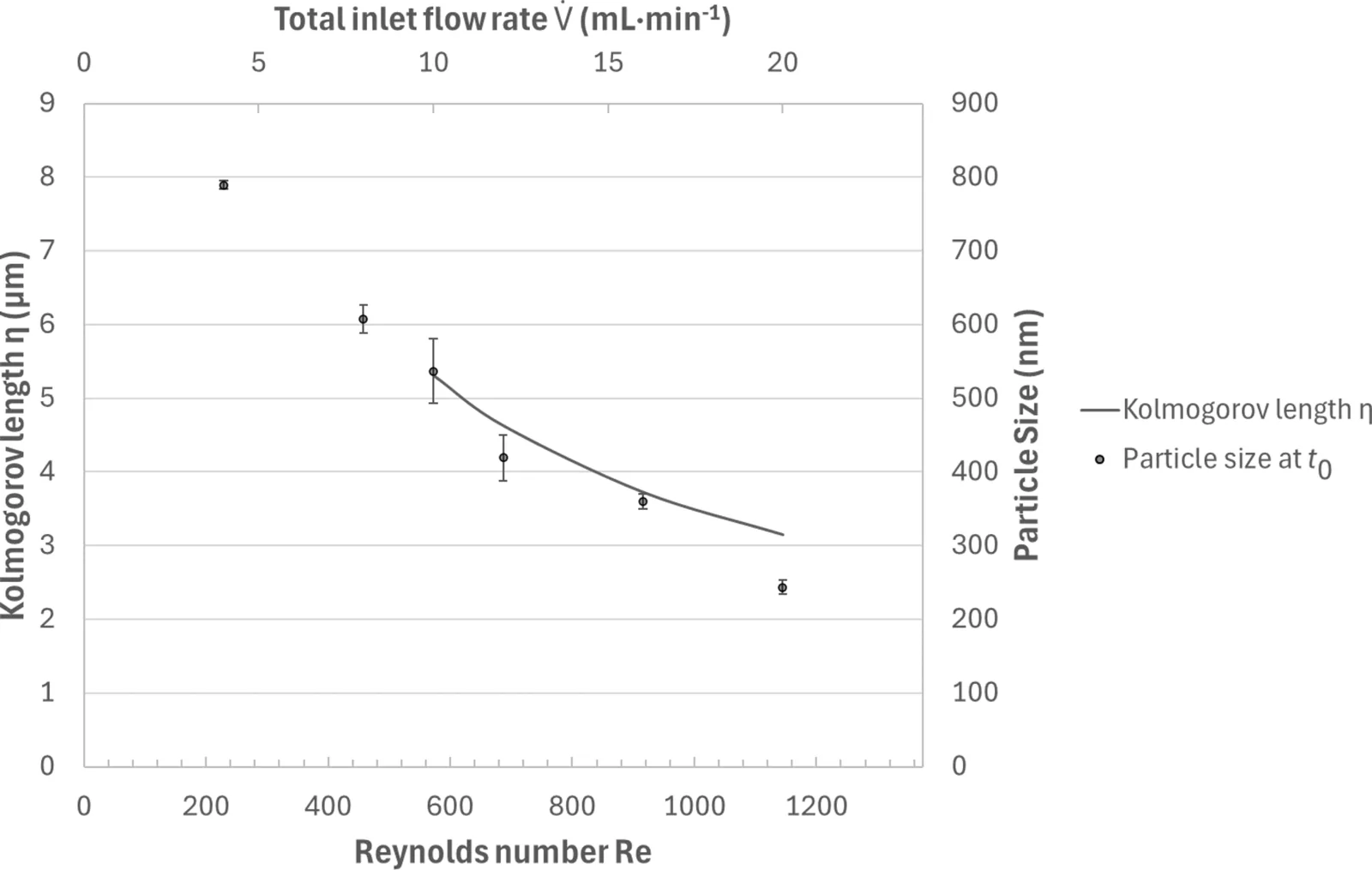
The Kolmogorov length η and the particle size at *t*_0_ as functions of the total inlet flow rates and Re without maturation section (*n* = 3).

Figure 3 illustrates the dependence of the Kolmogorov length η and the particle size at *t*_0_ on Reynolds number and total inlet flow rate. In the investigated range, the hydrodynamic conditions within the CIJM range from a purely laminar state (Re = 57–460 at total inlet flow rates between 1 and 8 mL·min^−1^) and the onset of incipient turbulence (Re = 573 at total inlet flow rate of 10 mL·min^−1^) to fully developed turbulence. This indicates that particle formation is governed by mixing-controlled nucleation and growth at high total inlet flow rates (≥10 mL·min^−1^): Smaller eddies promote more homogeneous supersaturation fields, leading to a larger number of nuclei and smaller final particles with narrower size distributions [21].

At low total flow rates (<4 mL·min^−1^), an unexpected increase in operational pressure was observed, approaching the system’s safety threshold (≈150 bar). This effect is attributed to local precipitation and particle deposition within the mixing zone, causing partial blockage of microchannels rather than purely hydrodynamic pressure buildup. Reduced shear and slower mixing at low total flow rates promote premature MnCO_3_ nucleation near channel walls, consistent with enhanced fouling in poorly mixed precipitation reactors, leading to clogging the pump and pipe system [29,32].

Collectively, these results highlight that flow rate in the CIJM is a critical design parameter: It controls Re, η, and mixing time, thereby determining nucleation density, MP size, and PDI. An intermediate total flow rate of 10 mL·min^−1^ was selected for all subsequent maturation experiments as a compromise between small particle size, low PDI, and safe, blockage-free operation.

### Hydrodynamics and transport in the maturation section

Following the mixing chamber, the suspension entered a straight polyetheretherketone (PEEK) capillary maturation section with an internal diameter of 0.75 mm (corresponds to 4.42 × 10^−7^ m^2^ cross section area). At total flow rate of 10 mL·min^−1^ (corresponds to 1.67 × 10^−7^ m^3^/s), the mean axial velocity (obtained by dividing the total flow rate by the cross-sectional area) was 0.38 m·s^−1^, corresponding to a tube Re near 300, that is, laminar flow [33–34]. In laminar Poiseuille flow, particles experience a parabolic velocity profile; however, finite-size and inertial effects cause lateral migration (Segré–Silberberg effect) [35,42]. Larger MPs migrate towards an annulus between the centerline and the wall, whereas smaller nuclei remain more diffusion-dominated [33–35,42]. Convective transport along the tube strongly exceeds molecular diffusion, but radial diffusion remains relevant for mass transport to growing particles and for smoothing concentration gradients inherited from the CIJM outlet [33–35]. The combined action of convection, inertia, and diffusion determines local supersaturation and collision frequencies along the tube and thus influences growth, aggregation, and protein adsorption dynamics [31–35].

The laminar flow in the maturation section does not generate any eddies and therefore does not cause shear-induced breakup of MPs; instead, it predominantly supports diffusive growth and interparticle interactions under relatively gentle shear [34,42]. Table 2 summarizes the effect of maturation section length and residence time τ_mature_ on HSA–MnCO_3_ MP properties. Under the applied experimental conditions, the encapsulation efficiency (EE%) increased markedly with τ_mature_, from 43% at τ_mature_ = 0 s to 65% at τ_mature_ = 12 s. The approximately 18% difference in HSA EE% was not accompanied by a measurable change in hydrodynamic particle size, indicating that differences in protein loading did not substantially affect the MnCO_3_ nucleation and particle growth; they primarily affected the internal composition of the MnCO_3_ particles. Instead, the change probably occurred at the level of protein incorporation through differences in protein–Mn^2+^ interactions [12], adsorption to surface of the primary MnCO_3_ nanoparticles and entrapment within their internal spaces or pores during the colloidal aggregation of the primary nanoparticles [43–44]. At τ_mature_ = 12 s, the EE% is comparable to the EE% from earlier experiments involving manual precipitation [9,12]. Simultaneously, the zeta potential became more negative with increasing τ_mature_. This shift indicates a higher contribution of proteins in the MP at longer residence times. This suggests that the maturation section primarily affects post-nucleation processes, such as protein adsorption onto the initially formed nanoparticles.

**Table 2:** Effect of maturation section and residence time τ_mature_ on encapsulation efficiency, particle size, PDI, and zeta potential (ZP) at 10 mL·min^−1^.

Maturation volume [mL]	Maturation length [m]	τ_mature_ [s]	EE%	mg HSA captured by 100 mg MnCO_3_	Particle size [nm]	PDI	ZP [mV]

0^a^	0.05^a^	0^a^	43.1% ± 2.0%	15.0 ± 0.7	568 ± 10	0.18 ± 0.02	−22.7 ± 0.2
1	2.26	6	63.2% ± 0.8%	22.0 ± 0.3	587 ± 67	0.15 ± 0.05	−24.8 ± 0.2
2	4.52	12	65.0% ± 0.2%	22.6 ± 0.1	567 ± 63	0.20 ± 0.02	−25.9 ± 0.2

^a^A 5 cm length of tubing was needed to collect the particles. The maturation volume and residence time were approximately 0.

Taken together, these findings reveal a functional separation of roles between the CIJM and the maturation section. The CIJM dictates nucleation density, particle size, and PDI via high-intensity micromixing and rapid supersaturation, driven by the strong kinetic energy dissipation at the impingement zone. The downstream laminar maturation section, in contrast, allows for fine-tuning of encapsulation efficiency and surface charge at almost constant size and PDI by controlling the time available for protein–crystal interactions under gentle shear and diminishing supersaturation. Such decoupling of size control (in the micromixer) and encapsulation tuning (in the maturation section) is highly advantageous for engineering hybrid MPs for drug delivery, where independent optimization of size, loading, and surface properties is often required [1–7,9–11,45].

## Conclusion

This work investigated the continuous co-precipitation of HSA–MnCO_3_ hybrid microparticles using CIJM combined with a tubular maturation section. By systematically varying flow rate in the mixing chamber and residence time downstream, we identified the distinct process parameters that govern particle formation and protein incorporation.

The results demonstrate that particle size and size distribution are predominantly controlled by the hydrodynamic conditions in the CIJM chamber. Increasing the total flow rate reduced the Kolmogorov length scale and promoted more efficient micromixing, yielding smaller microparticles. These findings confirm that nucleation and early growth occur within a mixing-dominated regime defined by the turbulent collision zone.

In contrast, extending the residence time in the maturation section had no substantial influence on particle size or PDI but strongly affected encapsulation efficiency. Longer residence times increased protein incorporation from 43% to 65% and shifted the zeta potential toward more negative values, indicating enhanced adsorption and integration of HSA during the post-mixing phase. Thus, maturation primarily modulates interfacial and compositional properties rather than particle dimensions.

These results highlight that CIJM enables reproducible microparticle formation driven by flow-controlled supersaturation, while the maturation section provides an effective means to tune protein loading and surface characteristics without compromising colloidal uniformity. This combination offers practical flexibility for producing hybrid protein–inorganic microparticles with properties relevant for drug delivery and related nanomedical applications.

## Experimental

### Materials

Manganese(II) chloride tetrahydrate (MnCl_2_·4H_2_O), sodium carbonate (Na_2_CO_3_) and human serum albumin (HSA) were purchased from Sigma-Aldrich Chemie GmbH (Taufkirchen, Germany). Isotonic saline solution (0.9% NaCl) was obtained from B. Braun SE (Melsungen, Germany). Hybrid HSA–MnCO_3_ MPs were synthesized using an IJM NanoScaler and associated software PurityChrom 6 (KNAUER Wissenschaftliche Geräte GmbH, Berlin, Germany), equipped with three high-pressure dosing pumps (model 80P), two computer-controlled valves, and interchangeable CIJM chambers. Polyetheretherketone (PEEK) capillary tubes (inner diameter 0.75 mm, 1/16″ outer fittings) were used as maturation sections and assembled with lengths of 0.05, 2.26, and 4.52 m.

### Methods

#### Fluid characterization

Solution densities were measured at room temperature using a pycnometer (Paul Marienfeld GmbH & Co. KG, Lauda-Königshofen, Germany). Dynamic viscosities were determined with a rotational rheometer (MCR 302, Anton Paar, Graz, Austria) by recording shear stress as a function of shear rate (1–1000 s^−1^); kinematic viscosity is calculated by dividing dynamic viscosity by fluid density.

#### Synthesis of HSA–MnCO_3_ microparticles and influence of flow rate on mixing and microparticle formation

Equimolar precursor stock solutions of 0.25 M Na_2_CO_3_ and 0.25 M MnCl_2_ containing 10 mg·mL^−1^ HSA were prepared in deionized water. Both stock solutions were fed to IJM NanoScaler.

To assess the impact of hydrodynamics in the CIJM, the total volumetric flow rate was varied between 1 and 20 mL·min^−1^, corresponding to different Reynolds numbers and turbulence intensities. For each condition, hydrodynamic quantities (Re, ε, η) were calculated using Equations 1–4, based on the measured fluid properties and chamber dimensions [25–30].


[1]

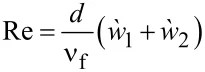




[2] $$\eta ={\left(\frac{\nu_{\text{f}}^{3}}{\varepsilon }\right)}^{1/4}$$

[3]

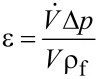




[4]

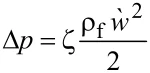



Here, Re is the Reynolds number of the colliding jets, 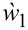 and 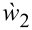 are the mean inlet velocities of the stock solutions of Na_2_CO_3_ and MnCl_2_ containing HSA, respectively, *d* is the inlet diameter (both inlets of the IJM chamber have the same diameter), ν_f_ is the kinematic viscosity (arithmetic mean, Table 1), η is the Kolmogorov length scale, ε is the energy dissipation rate per unit mass, 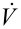 is the volumetric flow rate, *V* is the chamber volume, and ρ_f_ is the fluid density. The values of ε and, subsequently, η were calculated utilizing a resistance coefficient of ζ ≈ 0.69, which is characteristic for symmetrical T-shaped impinging jets [46].

For subsequent maturation experiments, a total flow rate of 10 mL·min^−1^ (5 mL·min^−1^ per inlet) was selected. Immediately downstream of the CIJM outlet, the suspension passed through a PEEK capillary maturation section of defined length (0.05, 2.26, or 4.52 m; internal diameter 0.75 mm). The corresponding hydraulic residence times τ_mature_ were calculated from Equation 5.


[5]

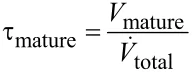



Here, *V*_mature_ is the volume of the maturation section and 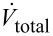 is the total volumetric flow rate.

After collection, the particles were incubated on a roller mixer for 30 min. The MPs were then washed three times with deionized water by centrifugation (3,000*g*, 5 min), and finally redispersed in 0.9% NaCl for further characterization.

#### Determination of the solids fraction of the particle dispersion

The particle suspension was centrifuged at 20,000*g* for 25 min, after which the supernatant was discarded. The wet particle pellet was dried at 45 °C. The solid content was calculated as a mass fraction by relating the mass of the dried particle pellet to the total mass of the original colloidal dispersion.

#### Determination of HSA concentration and encapsulation efficiency

The HSA concentration in supernatants was quantified by UV–vis spectrophotometry (Cary 60, Agilent Technologies, USA) at 280 nm, exploiting the absorbance of aromatic amino acids [16]. Calibration curves were generated from standard HSA solutions in the relevant concentration range.

Encapsulation efficiency (EE%) was calculated from the initial HSA mass *m*_HSA,0_ and the mass of non-encapsulated HSA in the supernatant *m*_HSA,_*_S_* after centrifugation:


[6] $$\text{EE}\%=\frac{m_{\text{HSA},0}-m_{\text{HSA},S}}{m_{\text{HSA},0}}\times 100$$

All measurements were performed in triplicate.

#### Particle size, polydispersity index, and zeta potential

The hydrodynamic diameter, polydispersity index, and zeta potential were determined by dynamic light scattering and laser Doppler electrophoresis using a Zetasizer Nano ZS (Malvern Instruments, UK).

#### Scanning electron microscopy

Samples were examined with a scanning electron microscope Digital Scanning Microscope DSM 950 (Zeiss, Germany) at an operation voltage of 10 kV.

## Data Availability

Data generated and analyzed during this study is available from the corresponding author upon reasonable request.
